# Rapid diagnostic tests failing to detect *Plasmodium falciparum* infections in Eritrea: an investigation of reported false negative RDT results

**DOI:** 10.1186/s12936-017-1752-9

**Published:** 2017-03-06

**Authors:** Araia Berhane, Mulugeta Russom, Iyassu Bahta, Filmon Hagos, Michael Ghirmai, Selam Uqubay

**Affiliations:** 1 Communicable Diseases Control Division, Ministry of Health, Asmara, Eritrea; 2 Eritrean Pharmacovigilance Center, Ministry of Health, Asmara, Eritrea; 3 National Medicine and Food Administration, Ministry of Health, Asmara, Eritrea; 4 Imaging and Laboratory Services Unit, Ministry of Health, Asmara, Eritrea; 5 Inspection Unit, National Medicine and Food Administration, Ministry of Health, Asmara, Eritrea; 6 National Malaria Control Programme, Ministry of Health, Asmara, Eritrea

## Abstract

**Background:**

Relatively large number of false-negative malaria rapid diagnostic test (RDT) results for microscopically confirmed *Plasmodium falciparum* cases were reported from five of the six administrative regions of Eritrea in 2015. This activated the Ministry of Health to conduct an initial exploratory investigation. The main objective of the investigation was to confirm the sensitivity of the RDTs in the field in microscopically confirmed malaria cases, identify the possible causes of the failure and recommend further actions to be taken.

**Methods:**

A team composed of the National Malaria Control Programme, National Medicines and Food Administration and Laboratory Unit of the Ministry of Health was established to confirm the sensitivity of the SD Bioline^®^ RDTs. A ‘Malaria RDT quality monitoring form’ was prepared and distributed to 13 health facilities selected on availability of microscopy services, experienced laboratory personnel and malaria endemicity, to carry out preliminary investigation on the suspected RDT quality defect. In parallel, field visits to central and regional medical warehouses as well as selected health facilities were conducted to assess the storage conditions, handling and operator procedures. Furthermore, joint field assessment was conducted with the manufacturer, SD Bioline RDTs. During the time frame of 15 July 2015 to 19 January 2016, 65 microscopically confirmed patients were tested with Malaria RDTs SD Bioline Pf/Pv/Mixed Combo cassettes.

**Results:**

A total of 65 blood specimens (50 *P. falciparum*, 13 *Plasmodium vivax* and 2 mixed) confirmed microscopically were tested against the available lots of malaria RDTs. Out of the 50 *P. falciparum* infected blood specimens, only 10 were confirmed positive with all the lots of *P*fHRP-2 detecting RDTs making the false negativity rate at 80% [41/51]. The false negative result for RDT targeting PfHRP2 antigen ranged from 65% [11/17] in Gash Barka region to 100% [12/12] in Northern Red Sea Region. In addition, supervisory visits undertaken by the study team have ruled out storage, handling and operator errors as causes of false negative results as the above parameter were found to be up to standards.

**Conclusion:**

The investigation confirmed high false-negativity rate in PfHRP-2 detecting RDTs and has ruled out quality of RDTs, storage, handling and operator error as causes of the reported problem. Therefore, molecular characterization of the *P. falciparum* is highly recommended to explore if parasite characteristics can be considered as causes of false negative results.

**Electronic supplementary material:**

The online version of this article (doi:10.1186/s12936-017-1752-9) contains supplementary material, which is available to authorized users.

## Background

Eritrea is a country located in the Horn of Africa, where 67% of its inhabitants live in malaria risk areas [1]. Though, malaria was one of the main public health problems in the country, intensive control interventions over the years, have resulted in marked decline in malaria cases and related deaths [2–4]. Integrated vector management, early diagnosis and treatment of malaria using parasitological confirmation at health facility and community levels were the main strategies deployed in the control of malaria. Based on the 2012 World Health Organization (WHO) malaria treatment guidelines [5], and the recommendation on malaria diagnosis in low-transmission settings [6], all suspected malaria cases were confirmed using microscopy or rapid diagnostic tests (RDTs). The use of microscopy in Eritrea is recommended for health facilities with laboratory services mainly at health centres and hospitals, and RDTs at health stations and community level. For more than 10 years, Eritrea has been using quality assured RDTs detecting *Plasmodium* antigens to diagnose malaria at health station level which gradually expanded to community health agents and drug retail outlets. Based on the health facilities report, the dominant species in Eritrea are *Plasmodium falciparum,* accounting for 67% of the confirmed malaria cases, followed by *Plasmodium vivax* for the remaining 30% and with few proportions of mixed infections [7]. Based on this distribution, RDTs detecting histidine-rich protein 2 and *Plasmodium* lactate dehydrogenase (*Pf*hrp2/pLDH) have been deployed to enable health providers detect all locally prevalent malaria species.

In accordance to the recommendations, RDTs were deployed in 2007 following training of health workers. These have been used with very good results and minimized the number of cases that were treated clinically at all levels and improved rational use of anti-malarial drugs. In the initial period, the type of RDTs that was deployed was of a *P. falciparum*-only type, which was later on replaced with tests detecting both *P. falciparum* and *P. vivax*. In most cases, the RDTs used were quality-assured before shipment.

Starting from mid-2015, the National Malaria Control Programme and the Eritrean Pharmacovigilance Center received from health facilities reports of suspected quality defects on the WHO Prequalified RDTs (SD Bioline Pf/Pv/Mixed Combo cassettes) that had previously passed WHO-sponsored pre-shipment quality testing. The facilities reported failures of RDTs detecting microscopically confirmed malaria cases. Initially there had been a few false-negative result complaints reported in late 2014, which were dismissed thinking that this could have been due to operator errors. However, in 2015, relatively large number of false-negative complaints from five of the six administrative regions activated the Ministry of Health to conduct initial investigation before contacting the manufacturer.

The main objective of the investigation was to confirm the sensitivity of the RDTs in the field in microscopically confirmed malaria cases, identify the possible causes of the failure and recommend further actions to be taken. Capacity of microscopic diagnosis of suspected malaria patients is available at health centres and hospitals, which are medium level and highest level health care delivery facilities, respectively. Refresher training is conducted annually on malaria microscopy to enhance capacity of laboratory technologists. Moreover, regular internal and external quality control systems are in place with higher level facilities (selected regional hospitals) cross-checking blood films from lower level facilities while the National Health Laboratory (NHL) cross-checks highly discrepant slides according to national guidelines. The NHL also participates in external quality control activities facilitated by NHLS/NICD based in South Africa. This paper focuses on the result of this investigation and the possible outcomes.

## Methods

A team composed of the National Malaria Control Programme (NMCP), National Medicines and Food Administration (NMFA) and Laboratory Unit of the Medical Services department was established to confirm the sensitivity of the SD Bioline RDTs. The team jointly prepared a ‘Malaria RDT quality monitoring form’ and distributed it to 13 health facilities (distributed in four of the six administrative regions) selected on availability of microscopy services, experienced laboratory personnel and malaria endemicity, to carry out preliminary investigation on the suspected RDT quality defect.

In the time-period 15 July 2015 to 19 January 2016, all patients who presented to the facilities with signs and symptoms of malaria, irrespective of age and sex, were eligible for inclusion for the study. Eligible patients were finger pricked for preparation of thin and thick blood films. All microscopically confirmed malaria patients were again finger pricked and tested with Malaria HRP2/P.f. HRP2/pLDH (SD Bioline Malaria Ag Pf/Pv) RDTs. Every health facility was instructed to test every microscopically malaria confirmed specimen against all the available lots of RDTs. The laboratory technologists were also oriented to document all laboratory test results and report both the blood film (B/F) and RDT results to NMFA or NMCP with proper labeling in the provided format. Thick and thin blood films were prepared and fixed with absolute methanol after which reading was conducted by expert microscopists. As part of quality control mechanism, all malaria positive slides were sent to the National Health Laboratory for second reading focusing on parasite identification and parasite count. The data collected from the investigation sites using structured format were entered into an Excel sheet within NMCP Office. Direct analysis on range of parasitaemia, percentage of false negative results by species and investigation site or zone was conducted.

**Fig. 1:**
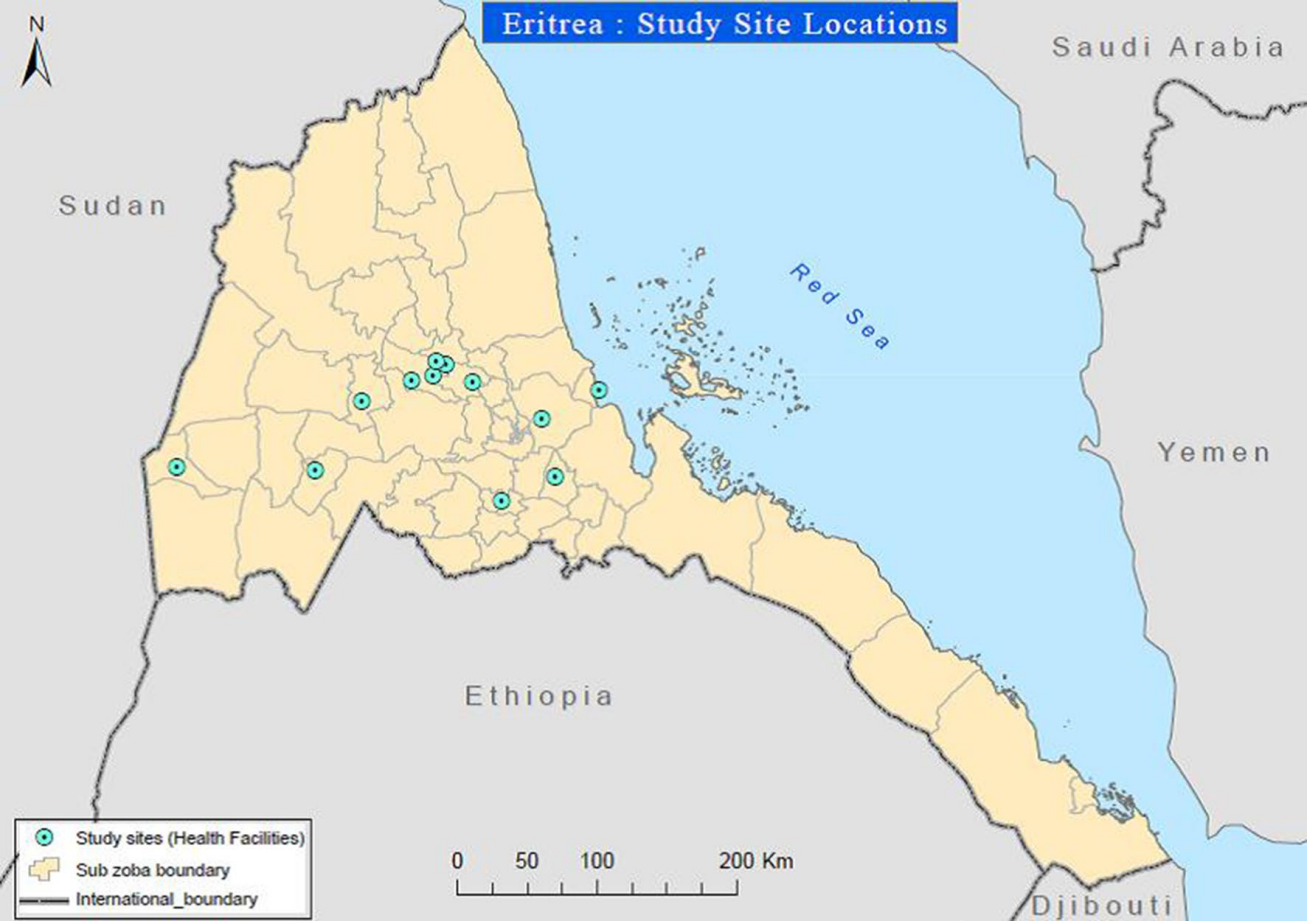
Study sites

The team also conducted assessment on the storage, handling and usage of RDTs and their shipment history to exclude external factors that can affect the performance. Moreover, to exclude environmental factors, 15 lots of RDTs retained in the Central Medical Store (PHARMECOR—Eritrea), an ideal warehouse, were distributed to the 13 designated study sites (Fig. 1).

In addition to the local assessment, joint assessment was conducted in-country with SD Company delegates on two types of blood: positive control prepared by SD at 2000 parasites/µl and blood taken from five malaria patients in two Hospitals of the country. Four of the specimens were fresh, while one was frozen for 3 days. In this joint assessment, four types of RDTs have been tested, namely SD Pf/Pv, SD Pf/Pan, CareStart^®^ Pf/Pv and RAPID 1-2-3^®^ Hema Cassette Malaria *Pf/Pv* (SD Bioline Malaria Ag Pf/Pv, SD, Bioline Malaria Ag Pf/Pa CareStart Malaria HRP2/PLDH (Pf/Pv) COMBO, and RAPID 1-2-3^®^ Hema Cassette Malaria PF/PV TEST, respectively) tests for comparative analysis. In addition, the SD *Pf/Pv* RDTs included those retained at SD Company (South Korea) and RDTs distributed to Eritrean health facilities.

### Ethical considerations

Consent was taken from all patients to provide blood sample for investigation. All confirmed patients were treated for malaria based on the national malaria treatment guideline.

### Limitations

The investigation was conducted as rapid response to the complaints repeatedly reported from the field and hence, sample size was not properly determined and the findings may not be representative of the situation in the whole country.

## Results

### Microscopy and RDT results

Out of the 13 health facilities designated to monitor the quality of malaria RDTs, 12 facilities located in four malarious regions of the country (Northern Red Sea, Anseba, Gash Barka & Debub) reported from which these data have been analysed. A total of 65 blood specimens (50 *P. falciparum*, 13 *P. vivax*, and 2 mixed) confirmed microscopically were collected and tested against the available lots of malaria RDTs. A total of 15 lots of RDTs were tested within the selected facilities.

**Fig. 2:**
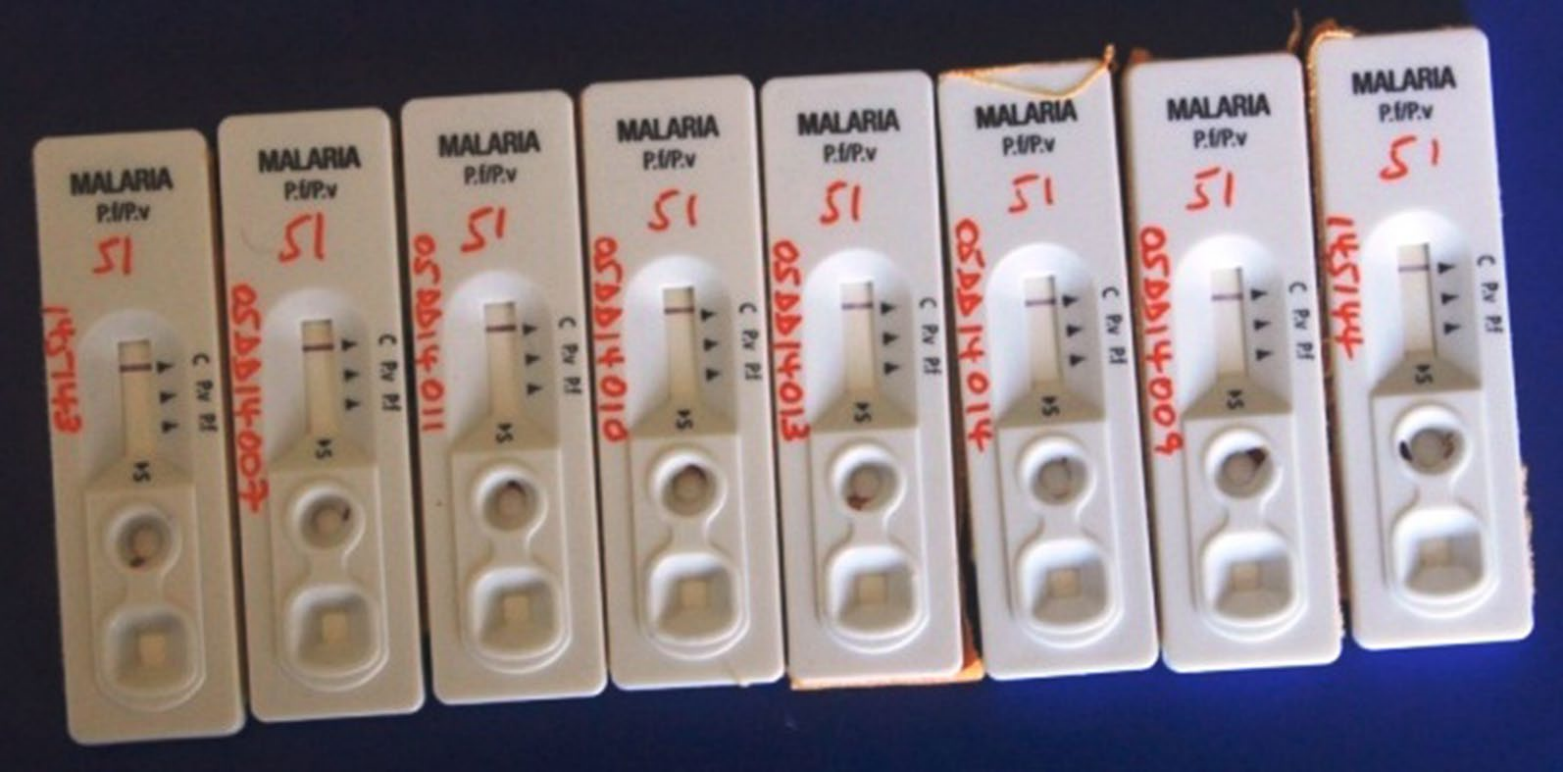
Some of the false negative results of malaria RDTs

The parasite density of the specimens ranged from 170 to 84,000 asexual parasites/µl with geometric mean of 10,094 parasites/µl. Out of the 50 microscopically confirmed *P. falciparum* infected blood specimens only 10 were confirmed positive with all the lots of RDTs tested making the false negativity proportion at 80% (41/51) (Fig. 2). The false negativity result for *Pf*HRP2 target antigen ranged from 61% (11/18) in Gash Barka region to 100% (12/12) in Northern Red Sea Region (Table 1). While the majority of *P. falciparum* infected blood specimens turned false negative when tested with RDTs, out of 13 *P. vivax* specimens only one tested false negative with RDTs. The *P. vivax* case that was false negative had low parasitaemia at 170/µl which could not be cross-checked at National Health Laboratory due to loss of the micro-slide. The two mixed infections, as confirmed by microscopy, were tested positive (*P. vivax)* for the first mixed infection and negative for the second mixed infection when tested by RDTs. The above RDT results were consistent across all lots of Pf/Pv (HRP/pLDH) RDTs tested at respective health facilities.

**Table 1:** Result of blood specimens tested microscopically and by RDT

Region	*P. falciparum*			*P. vivax*		
	Microscopically confirmed	Negative with RDT	%	Microscopically confirmed	Negative with RDT	%
Northern Red Sea	12	12	100.0	0	0	0
Anseba	14	13	92.9	3	1	33
Gash Barka	17	11	64.7	8	0	0
Debub	7	5	71.4	2	0	0
	50	41	80.4	13	1	7.7

### Joint assessment with the manufacturer

The joint assessment with the manufacturer of five *P. falciparum* patients yielded the following results. A known positive control, prepared at 2000 parasites/µl, gave positive results when tested against all SD Bioline HRP2Pf/Pv RDTs whether they were retained with the manufacturer or those distributed to the field in Eritrea. However, the five malaria patients (all were microscopy confirmed *P. falciparum*-infected) could not be detected by all the RDTs, except with SD Pf/Pan type where the Pan-line band only turned positive to each of the specimens (Table 2). All the microscopically confirmed cases were also re-confirmed by PCR by SD at the manufacturing facility.

**Table 2:** Malaria patients and test results of different RDT types, Ghindae Hospital

Specimen	Microscopy confirmation	SD Bioline HRP2Pf/Pv (retained at SD company)	SD Bioline HRP2Pf/Pv distributed to Eritrea health facilities	SD Bioline HRP2Pf/Pan	CareStart HRP2Pf/Pv	Hema diagnostics HRP2Pf/Pv
Positive control (2000 *Pf*. parasites/µl	Confirmed by SD as Pf (by PCR)	Positive	Positive	Not tested	Not tested	Not tested
Patient 1	*P. falciparum*	Negative	Negative	Positive	Negative	Negative
Patient 2	*P. falciparum*	Negative	Negative	Positive	Negative	Negative
Patient 3	*P. falciparum*	Negative	Negative	Positive	Negative	Negative
Patient 4	*P. falciparum*	Negative	Negative	Positive	Negative	Negative
Patient 5	*P. falciparum*	Negative	Negative	Positive	Negative	Negative

### Storage and handling

The team confirmed that temperature monitoring chart, RDTs storage conditions, test procedures, performance of laboratory technologists were appropriate. Moreover, it was confirmed that the product was shipped to the country in refrigerated containers which were electrified during travel as well as ports of transit and destination.

## Discussion

In Eritrea, RDTs have been the mainstay of malaria diagnosis in majority of health facilities and community health workers with no microscopy services. However, whenever three-quarters of the RDT tests fail to detect falciparum parasites, obviously it affects the confidence of health workers on this diagnostic method. Moreover, this has far reaching implications such as treating patients on clinical suspicion, threat of drug resistance, as well as possibility of missing a malaria case and consequent complications and mortalities [8]. This observed failure is divergent from the WHO minimum selection criteria for malaria RDTs, i.e. Panel Detection Score (PDS) against *P. falciparum* samples should be at least 75% at 200 parasites/µL [9].

Many factors affect the performance of RDTs starting from the quality of the product, its transportation, storage and distribution to the skill of the operator [8, 10, 11]. While the root cause analysis continues, findings of the initial investigation unambiguously highlighted that the suspected quality defect of the malaria RDTs or low parasite densities as cause to the false negativity are to be ruled out. This is evident from the fact that the product (SD Bioline Pf/Pv/Mixed Combo cassettes) was Prequalified by WHO have passed WHO-sponsored pre-shipment quality testing, have been shipped in refrigerated containers that were powered during shipment and ports of transit/destination and that local storage condition in the field were optimal.

The microscopy and PCR-confirmed *P. falciparum* samples were negative for *P. falciparum* test bands when examined with four similar types of RDTs (SD Pf/Pv, SD Pf/Pan, CareStart Pf/Pv and Rapid 1-2-3 Hema cassette malaria Pf/Pv test) showing that the problem was not limited to the SD Pf/Pv, but to all HRP-2 RDT brands. Neither SD Pf/Pv RDTs collected from the field nor those retained samples at SD Company resulted in positive bands for *P. falciparum*. However, the specimens tested positive for pan-line with SD Pf/Pan RDT, indicating that pLDH antigen has been detected. The *P. falciparum* samples that have been PCR-confirmed have also been shown to be negative for other plasmodium species as part of initial evidence and as recommended by Cheng et al. [12]. In addition, the false negative results for *P. falciparum* specimens occurred at low, medium and high parasite densities proving that parasitaemia did not influence the false negativity.

Besides to quality and handling of RDTs, parasite-related factors such as absence of antigens detected by these RDTs are also implicated for poor performance [12–14]. Such absence of PfHRP2 genes from *P. falciparum* was the cause of high false negativity in the Amazonian region of Peru [13], Mali [14] and recently in India [15]. The initial findings in this investigation i.e. negative result in all HRP-2 detecting RDT brands of three companies but positive in the SD Pf/Pan may be a clue that *Pfhrp2* antigen, which is the target for the *P*. *falciparum* band, might have been absent due to deletion of a gene that encodes the protein. Though, such type of gene deletion is not reported in the East African region, further molecular study is recommended to confirm presence or absence of the genes to guide future actions in the country. This investigation has provided health care professionals and malaria control programme an insight into effectiveness of malaria RDTs in different settings and its implication on treatment of suspected malaria cases.

## Conclusion

The currently prevailing situation of RDTs has certainly compromised malaria diagnosis and treatment in Eritrea, a country who has scored tremendous achievement in malaria control over the past 15 years. Analysis of the initial investigation of RDTs failing to detect malaria parasites has ruled out quality of RDTs, storage, handling and operator error as causes, paving the way for collecting confirmatory evidence on parasite related issues. Therefore, molecular characterization of the *P. falciparum* is highly recommended to examine parasite characteristics as causes of false negative results.

Important lessons have been learnt from the incidence under investigation, such that vigilance need to be heightened in monitoring the efficacy of RDTs regularly and that intermittent random check-ups must be conducted to cross-check the RDTs against other diagnostic tools such as light microscopy or PCR if feasible. Moreover, it is imperative that international guidance be produced on monitoring high false negativity result as it may have public health implication at local, regional and international levels.

## Additional file

**Additional file 1.** Annex 1: Results of internal assessment and Annex 2: Malaria Rapid Diagnostic Tests (RDT) quality monitoring form.

## Abbreviations

PfHRP2: *Plasmodium falciparum* histidine rich protein-2
pLDH: plasmodium lactate dehydrogenase
RDTs: rapid diagnostic tests
NMCP: National Malaria Control Programme
NMFA: National Medicines and Food Administration
