# Supplementary material for: Rapid diagnostic tests failing to detect Plasmodium falciparum infections in Eritrea: an investigation of reported false negative RDT results
Source: Malar J. 2017 Mar 6;16:105. doi: 10.1186/s12936-017-1752-9 (PMC5339986; doi:10.1186/s12936-017-1752-9)
Supplement: Supplementary file 1 — Additional file 1. Annex 1: Results of internal assessment and Annex 2: Malaria Rapid Diagnostic Tests (RDT) quality monitoring form. [file 12936_2017_1752_MOESM1_ESM.docx]

Additional file 1: Results of Internal Assessment

| **S.N** | **SITE** | **No. of PATIENTS EXAMINED** | ***P. falciparum*** | | | ***P. vivax*** | | |
| --- | --- | --- | --- | --- | --- | --- | --- | --- |
|  |  |  | **Microscopy Positive (Pf)** | **RDT Negative** | **%** | **Microscopy Positive (Pv)** | **RDT Negative** | **%** |
| 1 | Ghindae Hospital | 10 | 10 | 10 | 100 | 0 | 0 | - |
| 2 | Massawa Hospital | 2 | 2 | 2 | 100 | 0 | 0 | - |
|  | **Northern Red Sea Zone subtotal** | **12** | **12** | **12** | **100** | **0** | **0** | - |
| 3 | Keren Hospital | 2 | 2 | 2 | 100 | 0 | 0 | - |
| 4 | Hagaz Health Center | 3 | 1 | 1 | 100 | 1 | 0 | 0 |
| 5 | Elabered Health Center | 4 | 3 | 3 | 100 | 1 | 1 | 100 |
| 6 | Glas Health Station | 4 | 3 | 3 | 100 | 1 | 0 | 0 |
| 7 | St. George Health Station | 6 | 5 | 4 | 80 | 0 | 0 | **0.0** |
|  | **Anseba Zone subtotal** | **19** | **14** | **13** | **92.9** | **3** | **1** | **33.3** |
| 8 | Agordat Hospital | 8 | 7 | 8 | 100.0 | 1 | 0 | - |
| 9 | Barentu Hospital | 11 | 8 | 3 | 37.5 | 3 | 0 | 0.0 |
| 10 | Teseney Hospital | 6 | 2 | 0 | 0.0 | 4 | 0 | 0.0 |
|  | **Gash Baraka Zone subtotal** | **25** | **17** | **11** | **64.7** | **8** | **0** | **0.0** |
| 11 | Mendefera Hospital | 6 | 4 | 2 | 50.0 | 2 | 0 | 0.0 |
| 12 | Segheneiti Health Center | 3 | 3 | 3 | 100.0 | 0 | 0 | - |
|  | **Debub Zone subtotal** | **9** | **7** | **5** | **71.4** | **2** | **0** | **0.0** |
|  | Total | **65** | **50** | **41** | 80.4 | **13** | **1** | **7.7** |

| Additional file 2. Malaria Rapid Diagnostic Tests (RDT) Quality Monitoring Form | | | | | | | |
| --- | --- | --- | --- | --- | --- | --- | --- |
| Test No. | Test Date | Lot Number | Expiry Date | RDT Result (Neg/P.f/P.v/Mixed) | Microscopy Blood Film Result (Neg/P.f/P.v/Mixed) | Parasite density/µl | Remarks |
| 1 |  |  |  |  |  |  |  |
| 2 |  |  |  |  |  |  |  |
| 3 |  |  |  |  |  |  |  |
| 4 |  |  |  |  |  |  |  |
| 5 |  |  |  |  |  |  |  |
| 6 |  |  |  |  |  |  |  |
| 7 |  |  |  |  |  |  |  |
| 8 |  |  |  |  |  |  |  |
| 9 |  |  |  |  |  |  |  |
| 10 |  |  |  |  |  |  |  |
| 11 |  |  |  |  |  |  |  |
| 12 |  |  |  |  |  |  |  |
| 13 |  |  |  |  |  |  |  |
| 14 |  |  |  |  |  |  |  |
| 15 |  |  |  |  |  |  |  |
| 16 |  |  |  |  |  |  |  |
| 17 |  |  |  |  |  |  |  |
| 18 |  |  |  |  |  |  |  |
| 19 |  |  |  |  |  |  |  |
| 20 |  |  |  |  |  |  |  |
